# Functional characterization of SLC26A3 c.392C>G (p.P131R) mutation in intestinal barrier function using CRISPR/CAS9-created cell models

**DOI:** 10.1186/s13578-019-0303-1

**Published:** 2019-05-14

**Authors:** Nini Zhang, Daniel P. Heruth, Weibin Wu, Li Qin Zhang, Marianne N. Nsumu, Katherine Shortt, Kelvin Li, Xun Jiang, Baoxi Wang, Craig Friesen, Ding-You Li, Shui Qing Ye

**Affiliations:** 1 Division of Gastroenterology, Department of Pediatrics, Children’s Mercy Hospitals and Clinics, Kansas City, MO USA; 2 Division of Experimental and Translational Genetics, Department of Pediatrics, Children’s Mercy Hospitals and Clinics, Kansas City, MO USA; 3 Department of Biomedical and Health Informatics, University of Missouri Kansas City School of Medicine, Kansas City, MO USA; 4 Department of Pediatrics, Tangdu Hospital, Fourth Military Medical University, Xi’an, Shaanxi China; 5 Department of Biomedical Sciences, University of Missouri Kansas City School of Medicine, Kansas City, MO USA; 6 Division of Cell Biology & Biophysics, University of Missouri Kansas City School of Biological Sciences, Kansas City, MO USA; 7 Department of Global Biostatistics and Data Science, Center for Bioinformatics and Genomics, Tulane University, New Orleans, LA USA; 8 Department of Neonatology, Nanfang Hospital, Southern Medical University, Guangzhou, China

**Keywords:** Single-nucleotide polymorphism (SNP), Chloride transport, Epithelial cell, Inflammation, Intestinal epithelium

## Abstract

**Background:**

Congenital chloride diarrhea (CCD) in a newborn is a rare autosomal recessive disorder with life-threatening complications, requiring early diagnostics and treatment to prevent severe dehydration and infant mortality. SLC26A3 rs386833481 (c.392C>G; p.P131R) gene polymorphism is an important genetic determinant of CCD. Here, we report the influence of the non-synonymous SLC26A3 variant rs386833481 gene polymorphism on the function of the epithelial barrier and the potential mechanisms of these effects.

**Results:**

We found that P131R-SLC26A3 increased dysfunction of the epithelial barrier compared with wild type SLC26A3 in human colonic Caco-2 and mouse colonic CMT-93 cells. When P131R-SLC26A3 was subsequently reverted to wild type, the epithelial barrier function was restored similar to wild type cells. Further study demonstrated that variant P131R-SLC26A3 disrupts function of epithelial barrier through two distinct molecular mechanisms: (a) decreasing SLC26A3 expression through a ubiquitination pathway and (b) disrupting a key interaction with its partner ZO-1/CFTR, thereby increasing the epithelial permeability.

**Conclusion:**

Our study provides an important insight of SLC26A3 SNPs in the regulation of the epithelial permeability and indicates that SLC26A3 rs386833481 is likely a causative mutation in the dysfunction of epithelial barrier of CCD, and correction of this SNP or increasing SLC26A3 function could be therapeutically beneficial for chronic diarrhea diseases.

## Introduction

Globally, diarrhea is a leading cause of death among all ages (1.31 million deaths in 2015), as well as a leading cause of the disability-adjusted life years (DALYs) because of its disproportionate impact on young children (71.59 million DALYs, range from 66.44 million to 77.21 million), especially in developing countries [1]. The etiology and underlying molecular pathogenesis of diarrhea are complicated and not fully understood though it is increasingly recognized that genetic predisposition may play a significant role in an individual’s susceptibility to chronic diarrhea [2, 3]. Understanding the genetic contributions to disease biology can help identify at-risk individuals, guide more effective personalized treatment approaches, and illuminate new targets and pathways for therapeutic development and intervention.

Chronic diarrhea diseases can be classified into inflammatory, malabsorptive, osmotic, secretory and motility disorders [4]. Congenital chloride diarrhea (CCD-OMIM 214700) is an autosomal recessive disorder characterized by life-long, severe diarrhea with intestinal Cl^−^ malabsorption. Postnatal clinical diagnosis is based on the presentation of dehydration and failure to thrive in the setting of hypokalemic metabolic alkalosis, with acidic stool pH and elevated stool chloride (> 90 mM) measured after normalization of systemic volume status and serum electrolytes. Untreated disease leads to chronic systemic volume depletion, nephrocalcinosis and impaired renal function sometimes progressing to end-stage renal disease. Additional clinical manifestations later in life have included intestinal inflammation, hyperuricemia, inguinal hernia, and impaired male fertility [3].

CCD is caused by mutations in the gene encoding SLC26A3 [3, 5, 6], with 21 exons spanning ~ 38 kb on chromosome7q31.1. SLC26A3 is a Cl^−^/HCO_3_^−^ exchanger that contributes to intestinal fluid absorption and enterocyte acid/base balance [7, 8], which has been unequivocally demonstrated to be a Cl^−^/HCO_3_^−^ exchanger with a 2:1 transport stoichiometry [9, 10]. The transport function of SLC26A3 is thought to play an important role in Cl^−^ absorption and HCO_3_^−^ secretion in the colon and perhaps in the pancreas [11, 12].

Single nucleotide polymorphisms (SNPs) are the most common type of genetic variation among humans. Some SNPs have been proven to be directly associated with human diseases. SNPs that lead to amino acid substitutions in proteins are of particular interest because they are responsible for nearly half of the known genetic variations related to inherited diseases in human [13, 14]. Previous studies have found a number of SNPs in SLC26A3, including the damaging missense mutation rs386833481 (c.392C>G; p.P131R), from patients with CCD [3]. Whether any of these SNPs is a causative mutation has been unproven. Furthermore, patients with diarrhea associated with inflammatory bowel disease (IBD), either ulcerative colitis (UC) or Crohn’s disease (CD), exhibit reduced SLC26A3 expression [15, 16]. Xiao et al. [17] previously identified that SLC26A3 deficiency is associated with the absence of a firmly adherent mucus layer and mucus barrier impairment in mice. This change in mucus layer renders SLC26A3^−/−^ mice susceptible to dextran sulfate sodium (DSS)-induced colitis. These studies imply reduced SLC26A3 expression, leads to increased dysfunction of the epithelial barrier. However, little is known about whether these genetic variants could lead to the dysfunction of the epithelial barrier and about the potential mechanisms of these effects.

SLC26A3 interacts with cystic fibrosis transmembrane conductance regulator (CFTR) and they reciprocally regulate each other through binding of the R domain of CFTR and the STAS domain of SLC26A3 [18, 19]. Meanwhile, there is an increased permeability of the small intestine both in CF humans and in CF mice (*Cftr* knockout mouse model) [20], and CFTR interacts with ZO-1 to regulate tight junctions [21]. The importance of both SLC26A3 and CFTR functions in the physiology of tight junctions (TJs) is supported by their molecular interaction. These findings prompted us to study whether SNPs in SLC26A3 disturb its normal interaction with ZO-1/CFTR and increase intestinal epithelial permeability.

In this study, we dissected the functional consequences of the P131R variant and SLC26A3 expression level on intestinal epithelial permeability and functionally characterized the interaction between SLC26A3 SNP encoded protein or WT SLC26A3 protein and ZO-1/CFTR in human colonic Caco-2 cells. Further, we evaluated the therapeutic potential of correcting this SNP mutation of SLC26A3 by testing the function of epithelial barrier of Caco-2 cells. Our study provides solid evidence that SLC26A3 SNP rs386833481 (c.392C>G; p.P131R) is a likely causative mutation in the dysfunction of epithelial barrier of CCD. Our biochemical study has also provided a lead to the underlying molecular mechanism.

## Results

### Construction of the P131R-SLC26A3 genetic variant

**Fig. 1:**
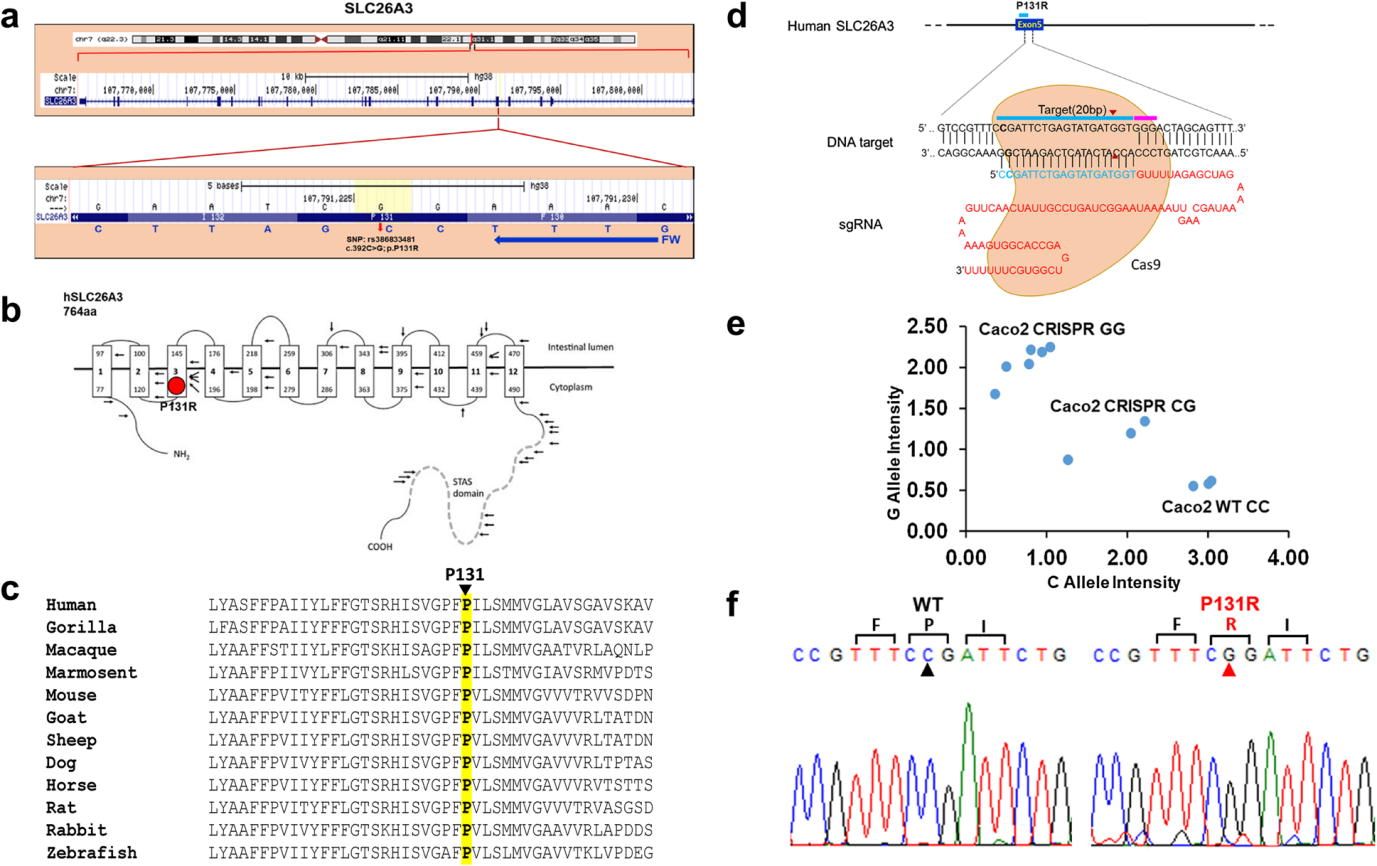
Construction and expression of P131R-SLC26A3 genetic variant on Caco-2 cells. **a** The SNP rs386833481 in the coding sequence of the SLC26A3 gene leads to the Proline to Arginine amino acid change at position 131. **b** Topographic model of hSLC26A3 (reproduced from Wedenoja et al. [3]) showing the predicted location of P131R within the transmembrane domain. **c** Alignment of mammalian SLC26A3 polypeptide sequences in the region of hSLC26A3 P131R (Highlight), showing completely conservation among species orthologs (CLUSTAL 2.1-multiple sequence alignment). **d** Schematic of the RNA-guided Cas9 nuclease. The Cas9 nuclease from *S. pyogenes* (in yellow) is targeted to human SLC26A3 P131R locus by a sgRNA consisting of a 20-nt guide sequence (blue) and a scaffold (red). The guide sequence pairs with the DNA target (blue bar on top strand), directly upstream of a requisite 5′-NGG adjacent motif (PAM; pink). Cas9 mediates a DSB ~ 3 bp upstream of the PAM (red triangle). **e** Results of TaqMan™ Genotyping Assay of DNA isolated from either wild-type Caco-2 cells (CC) or CRIPSR/Cas9 gene edited cells: (CG) and (GG). CRISPR/Cas9 transfected Caco-2 cells were selected with puromycin. Three CRISPR/Cas9n-Caco-2 puromycin resistant lines were tested. N = 3 TaqMan Assays per sample. **f** Results of Sanger sequencing for confirming the construction of P131R-SLC26A3 on Caco-2 cells by CRISPR/Cas9

Based on analysis of public databases, we identified an exonic SNP in the human SLC26A3 gene from patients with CCD. The SLC26A3 genetic variant (rs386833481) changes the DNA from a cytosine (C) to a guanine (G) base and an amino acid change from Proline (P) to Arginine (R) at its amino acid sequence position 131 (Fig. 1a). In this study, the SLC26A3 rs386833481 is referred to as P131R-SLC26A3. The P131R mutation was predicted to be “deleterious” and “damaging” by Provean (score − 7.32; cutoff: − 2.5) and Sift (score 0.001; cutoff: 0.05) web server tools for predicting the functional effect of amino acid substitutions. Amino acid residue P131 resides within the polytopic transmembrane domain of SLC26A3 (Fig. 1b). Although the membrane domains of SLC26 polypeptides are of unknown topographical disposition, hydropathy profiling has predicted a location for P131 at the putative transmembrane span3. This residue is conserved among SLC26A3 orthologs in primates, rodents, goat, sheep, dog, horse, rabbit and zebrafish (Fig. 1c). Until now, there is little information and indication of this SLC26A3 genetic variant being linked to human diarrhea susceptibility. To further explore whether the SLC26A3 genetic variant alters its function and expression, we adapted an HDR-mediated modification strategy using the CRISPR/Cas9 system in both human (Caco-2, Fig. 1d) and murine colonic epithelial (CMT-93, Fig. 6a) cell lines. After the SLC26A3 c.392C>G (p.P131R) mutation was generated in both cell lines, they went though a week-long puromycin selection for a single clone that carries the exact mutation. TaqMan SNP Genotyping (Fig. 1e) and Sanger Sequencing (Fig. 1f) both were used to validate the accurate construction of P131R-SLC26A3. These results indicated that we successfully recreated SLC26A3 SNP rs386833481 (c.392C>G; p.P131R), providing the foundation for functional analysis of its effect on intestinal epithelial cell permeability.

### P131R-SLC26A3 weakens the epithelial barrier and augments TNF-α-induced damage

**Fig. 2:**
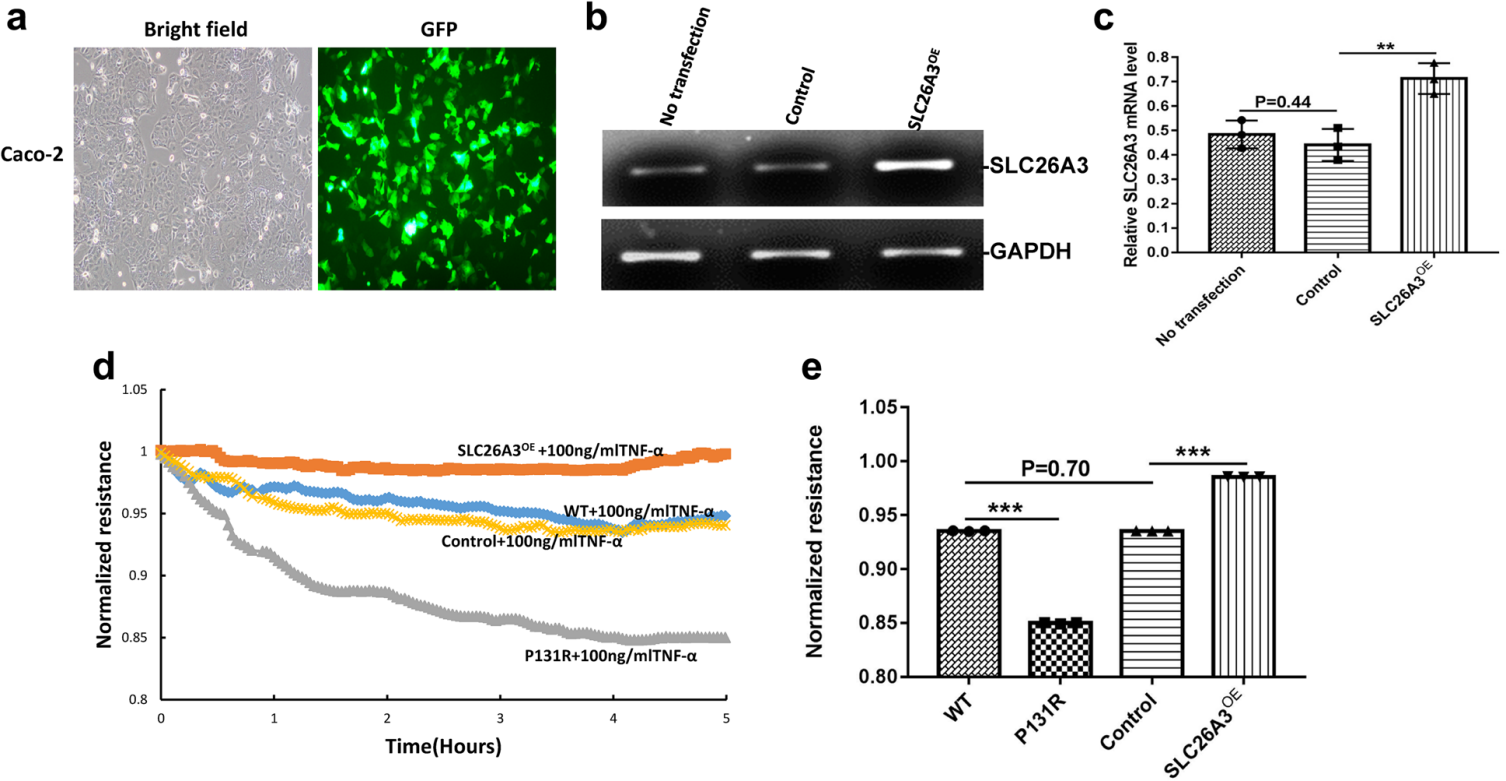
P131R-SLC26A3 weakens the epithelial barrier and augments TNF-α-induced damage. **a** Representative GFP for overexpressing SLC26A3. **b** Representative images of SLC26A3 mRNA levels in Caco-2 cells transfected with pCS6 (empty vector) and SLC26A3-pCS6 (SLC26A3^OE^) measured by RT-PCR. GAPDH expression was presented as control. **c** Relative RT-PCR quantification of SLC26A3 mRNA levels in Caco-2 cells transfected with pCS6(empty vector) and SLC26A3^OE^. **d** Caco-2 cells onto array chambers containing 40 gold electrodes per well (8W10E+) pretreated with 10 mM cysteine and coated with fibronectin (20 µg). The experiments were initiated when the cells reached confluence, as determined by a capacitance of 10 nF at 32,000 Hz, the monolayers were starved from serum for 2 h, then treated with 100 ng/ml of TNF-α. Representative figure of transepithelial electric resistance (TEER) data from ECIS analysis at 500HZ of wild-type Caco-2 cells, Caco-2 cells containing the rs386833481(c.392C>G; p.P131R) SNP generated by CRISPR/Cas9 knock-in, SLC26A3 overexpressing Caco-2 cells (SLC26A3^OE^) and Caco-2 cells transfected with empty vector pCS6 (Control) are shown. **e** P131R SLC26A3 in Caco-2 cells significantly decreased the maximum TNF-α-induced decrease in TEER value compared with WT Caco-2 cells, but SLC26A3 over expression prevented the TNF-α-induced decrease in TEER value compared with the empty vector pCS6(Control) cells. TNF-α treatment resulted in similar decrease of TEER values between WT and control cells. *P < 0.05, **P < 0.01, ***P < 0.001

To determine the role of P131R-SLC26A3 in epithelial barrier function, we also upregulated SLC26A3 expression by transfecting Caco-2 cells with either SLC26A3-pCS6 or the empty vector pCS6 control (Fig. 2b, c). A GFP expression vector was used to monitor transfection efficiency (Fig. 2a). Previous work showed that SLC26A3 expression is down-regulated in a TNF-α overexpressing mouse model and that TNF-α can affect the expression of tight junction proteins [22, 23]. We therefore measured transepithelial electric resistance (TEER) in P131R-SLC26A3, SLC26A3-overexpressing and normal Caco-2 cells. Upon TNF-α treatment, P131R-SLC26A3 cells showed significantly lower TEER values compared with normal cells. Consistent with these results, the TEER value in SLC26A3-overexpressing (SLC26A3^OE^) cells was higher than that in control cells (Fig. 2d). The maximum TEER induction are 0.85 ± 0.006 vs. 0.94 ± 0.003 in P131R-SLC26A3 vs. WT (P < 0.001), as well as 0.99 ± 0.003 vs. 0.95 ± 0.001 in SLC26A3^OE^ vs. control cells (P < 0.001), respectively. There was no significant difference between WT and control cells (P = 0.70) (Fig. 2e). These results indicated that P131R-SLC26A3 weakened the epithelial barrier and augmented TNF-α-induced damage. Further, overexpression of SLC26A3 prevented TNF-α induced epithelial barrier dysfunction.

### P131R-SLC26A3 is involved in the [Cl^−^]_i_ decrease induced by Tenidap and the epithelial barrier dysfunction induced by osmotic stress

**Fig. 3:**
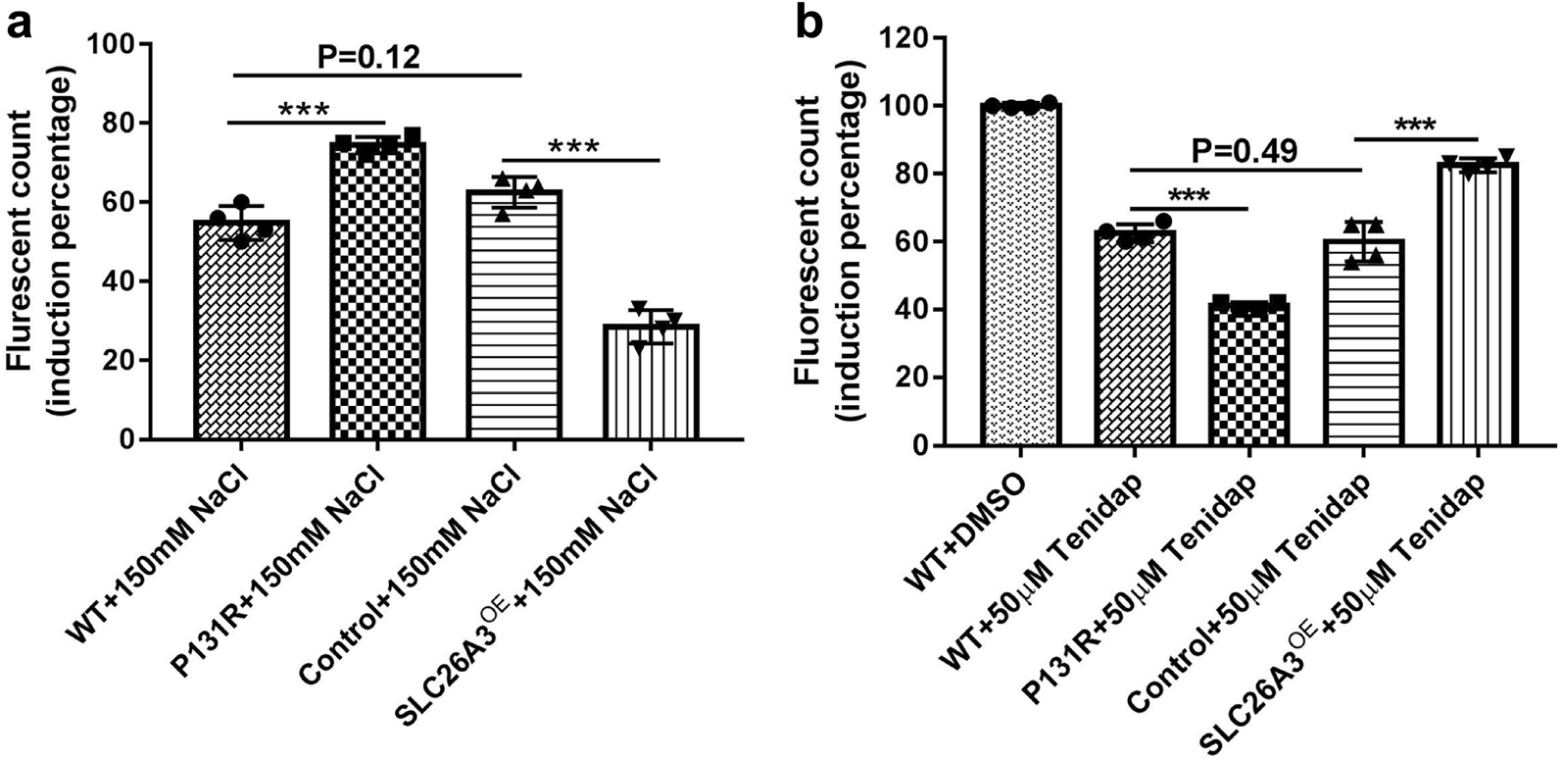
P131R-SLC26A3 is involved in the intestinal barrier dysfunction induced by osmotic stress and the [Cl^−^]_i_ decrease induced by Tenidap. **a** Caco-2 cells were seeded and cultured in growth medium until a monolayer was formed, the monolayers were starved for 1 h with Hank’s balanced salt solution (HBSS), then treated with HBSS or 150 mM of NaCl in HBSS. P131R-SLC26A3 significantly increased the 150 mM NaCl-induced increase fluorescence value (485/535 nm excitation/emission) compared with WT Caco-2 cells (P = 7.4 × 10^−4^), but SLC26A3 over expression prevented the NaCl-induced increase in fluorescence value compared with the empty vector pCS6 (Control) cells. NaCl treatment resulted in similar increase of fluorescence values between WT and control cells (N = 4). **b** Intracellular Cl^−^ measurements with MQAE (10 mM) were performed in CRISPR/Cas9 edited Caco-2 cells. SLC26A3 inhibitor Tenidap (50 µM) significantly decreased [Cl^−^]_i_ in P131R-SLC26A3 compared with WT cells. While SLC26A3 over-expressing cells partly prevented decrease of [Cl^−^]_i_ induced by Tenidap compared with empty vector (pCS6) control cells (N = 4). *P < 0.05, **P < 0.01, ***P < 0.001

Previously, Chávez et al. [24] reported that db-cAMP elevated [Cl^−^]_i_ in noncapacitated sperm, but this increase was inhibited by Tenidap, a SLC26A3 antagonist [25, 26]. Since P131R-SLC26A3 weakened the epithelial barrier, we explored whether this SNP was involved in the [Cl^−^]_i_ decrease induced by Tenidap. Intracellular Cl^−^ measurements with MQAE (10 mM) revealed that treatment with Tenidap (50 µM) significantly decreased [Cl^−^]_i_ in both P131R-SLC26A3 (42 ± 1%) and WT (63 ± 2%) cells compared to the DMSO treated controls (100 ± 1%; P < 0.001) (Fig. 3b). To investigate the function of SLC26A3 in response to hyperosmotic stress, we exposed confluent monolayers of Caco-2 cells to 150 mM sodium chloride (NaCl). The treatments provoked increased epithelial barrier dysfunction in P131R-SLC26A3 cells (75% ± 5%) compared with WT cells (56% ± 4%, P < 0.001), while overexpression of SLC26A3 lowered epithelial barrier dysfunction (29 ± 2%) compared with pCS6 vector control cells (62 ± 4%; P < 0.001) (Fig. 3a). These results indicated that P131R-SLC26A3 was involved in the [Cl^−^]_i_ decrease induced by Tenidap and the epithelial barrier dysfunction induced by osmotic stress.

### Correction of P131R-SLC26A3 to WT restored the epithelial barrier function

**Fig. 4:**
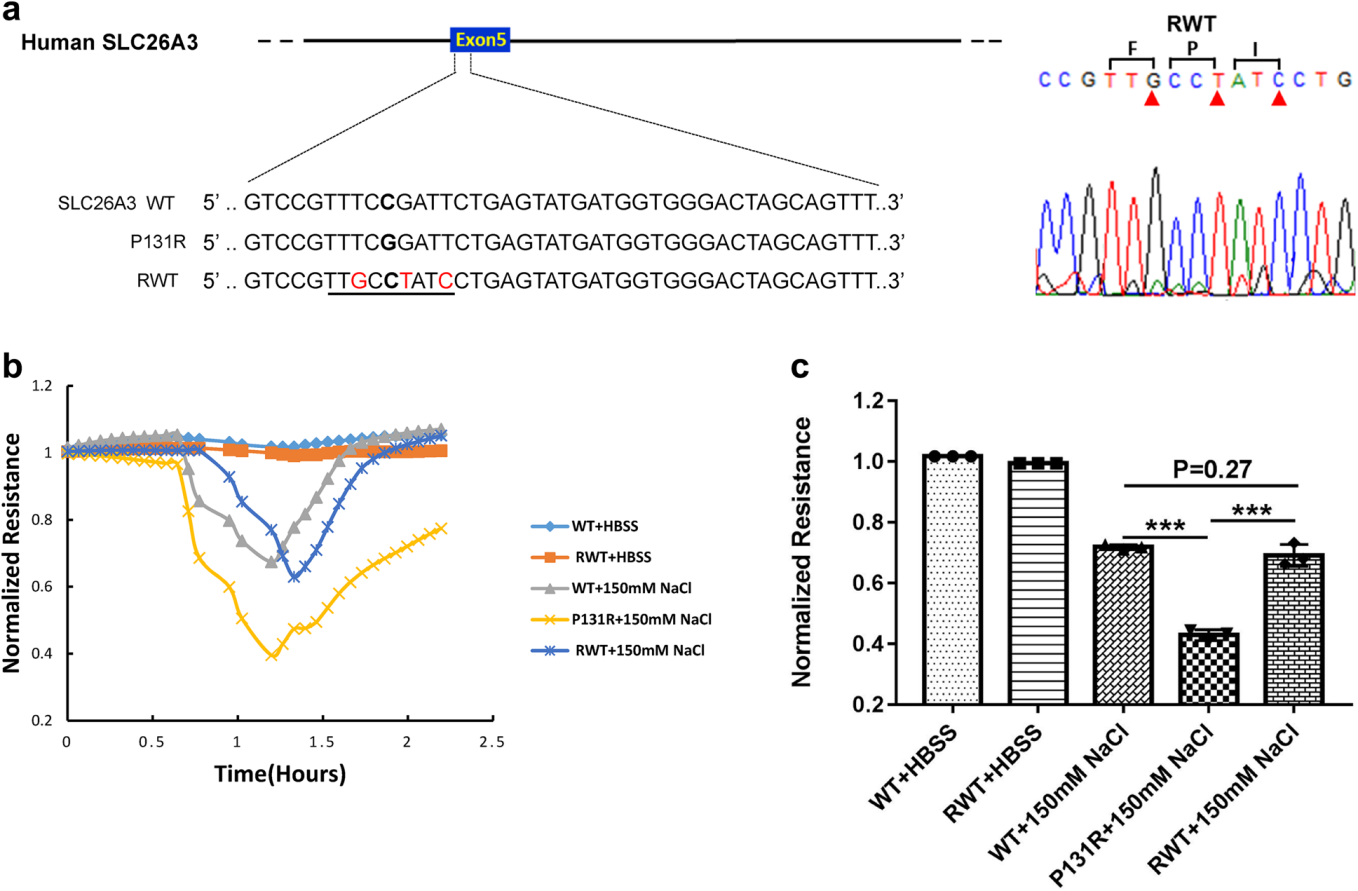
Functional analysis of the restored SLC26A3 function in reverted cells. **a** Sanger sequencing for confirming the correction of P131R SNP. We used a novel ssODN that codes for the same amino acids of WT SLC26A3, but uses unique codons for the F-P-I triAA sequence flanking the wild-type Proline that was changed to Arginine and now back to Proline. **b** TEER data from ECIS analysis of wild-type Caco-2 cells, Caco-2 cells containing the P131R and corrected cells (RWT). **c** Results from each group are presented as mean ± SD of three samples from three separate experiments. When SLC26A3-P131R was reversed back to wild type with the corrective donor templates by delivering Cas9/sgRNA vectors, a similar TEER and epithelial barrier function was observed in reverted cells. *P < 0.05, **P < 0.01, ***P < 0.001

To investigate if normal function of SLC26A3 could be restored by changing the P131R-SLC26A3 sequence back to a WT sequence, we employed CRISPR/Cas9 gene editing using a novel ssODN in Caco-2 cells. We designed an ssODN that coded for the WT-SLC26A3, but utilized unique codons for the three amino acid sequence (F-P-I) that spanned the wild-type Proline. This allowed us to differentiate the newly constructed WT gene from the original gene. Sanger sequencing validated the correction of P131R-SLC26A3 to WT-SLC26A3 (Fig. 4a). In order to investigate the function of SLC26A3 corrected P131R-SLC26A3 (RWT) cells, we exposed confluent monolayers of Caco-2 cells to 150 mM NaCl. The treatments provoked reduction in TEER in P131R-SLC26A3 cells relative to WT-SLC26A3 cells that indicated a significant decrease in epithelial barrier function (Fig. 4b). However, when SLC26A3-P131R was reversed back to wild type a similar TEER and epithelial barrier function was observed in the corrected cells (Fig. 4b). The maximum TEER inductions were 0.43 ± 0.02 vs. 0.72 ± 0.01 in P131R-SLC26A3 vs. WT (P < 0.001) and 0.69 ± 0.04 vs. 0.72 ± 0.01 in RWT vs. WT (P = 0.27) (Fig. 4c). These effects were transient and did not induce significant cellular loss, because TEER values recovered after withdrawal of the osmotic challenge. These results indicated that reverting P131R-SLC26A3 to WT can restore the epithelial barrier function.

### ZO-1/CFTR mediates the epithelial barrier dysfunction induced by TNF-α and osmotic stress and P131R-SLC26A3 promotes SLC26A3 ubiquitination

**Fig. 5:**
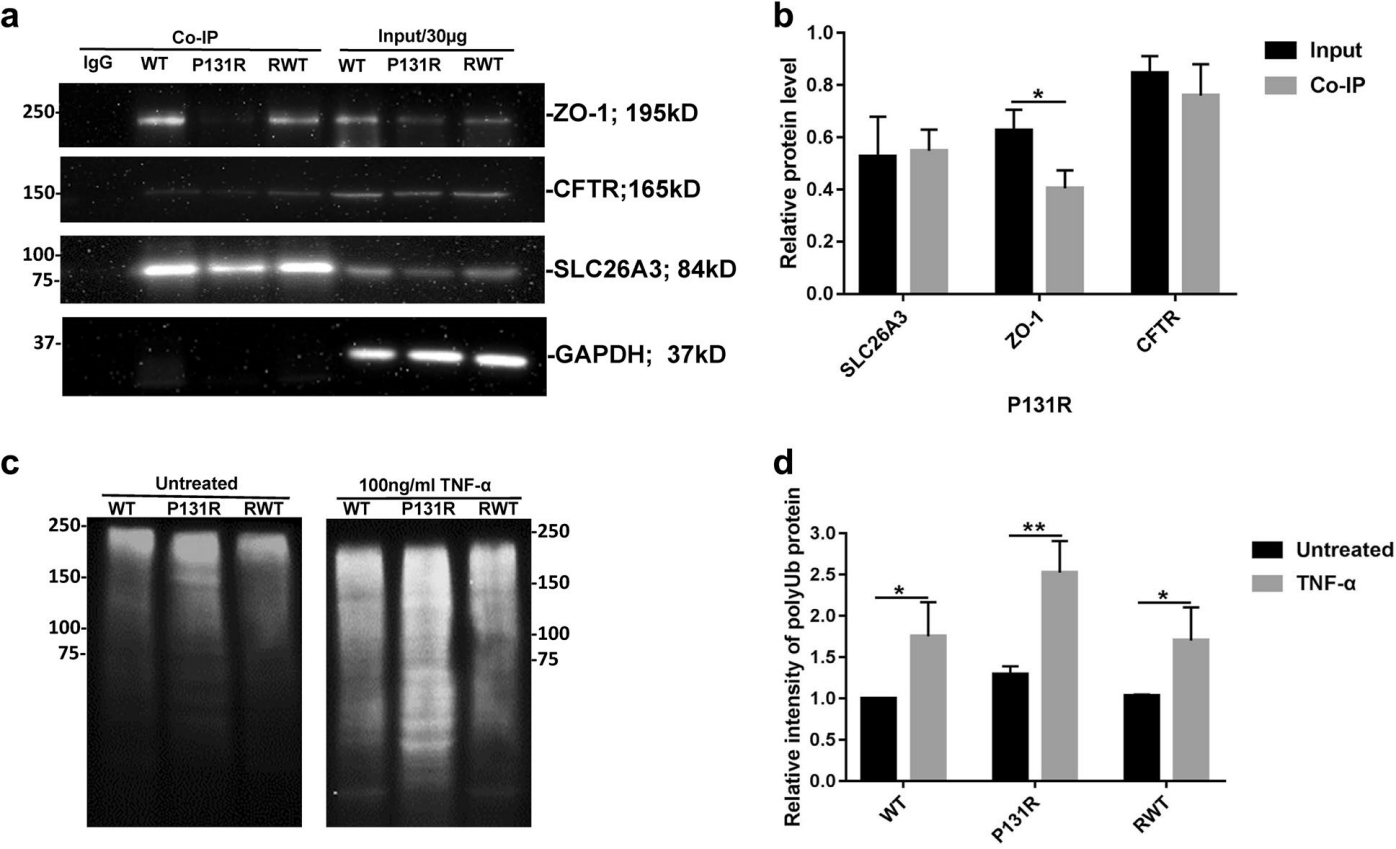
Identification of SLC26A3 as a CFTR and ZO-1 binding partner and effects on their interaction. **a** Endogenous IP assays of Caco-2 WT, P131R and RWT cell lysates showing that SLC26A3 co-precipitated with ZO-1 and CFTR, as well as that the interaction between SLC26A3 and ZO-1 is significantly decreased in P131R-SLC26A3 cells (*P *< 0.05). IgG was used as a negative control, GAPDH was used as an input control. **b** Densitometry analysis of endogenous IP assays. **c** P131R promotes SLC26A3 ubiquitination. WT, P131R and RWT cells were untreated (left panel) or stimulated with 100 ng/ml of TNF-α for 20 h (right panel), and cell lysates were assayed for immunoprecipitation (*IP*), and levels of ubiquitinated SLC26A3 were assessed by immunoblotting (*IB*). **d** Ratio of ubiquitination of protein between control and TNF-α treated groups. *P < 0.05, **P < 0.01, ***P < 0.001

Since TJ disruption is considered a vital event in the pathogenesis of intestinal inflammation, and there is increased permeability of the small intestine both in CF humans and in CF mice (Cftr knockout mouse mode) [20], we explored the physiological functions of P131R-SLC26A3 on the TJ protein ZO-1 and CFTR. Endogenous co-IP assays revealed that ZO-1 was immune-precipitated by the SLC26A3 antibody. In addition, SLC26A3 and ZO-1 protein levels are also decreased in P131R-SLC26A3 cells. Moreover, the interaction between SLC26A3 and ZO-1/CFTR are both decreased (Fig. 5a, b). To examine the detailed mechanism by which P131R-SLC26A3 induced lower levels of SLC26A3, we detected SLC26A3 degradation changes. As shown in Fig. 5c, d, P131R-SLC26A3 in Caco-2 cells resulted in increased ubiquitination of SLC26A3, which was intensified by TNF-α treatment. Correction of P131R-SLC26A3 to WT displayed similar ubiquitination status as the original WT. These results indicated that ZO-1/CFTR mediated the epithelial barrier dysfunction induced by TNF-α and osmotic stress, and that P131R-SLC26A3 further promoted SLC26A3 ubiquitination.

### Construction and function of Slc26a3 P131R genetic variant on murine colonic epithelial cells

**Fig. 6:**
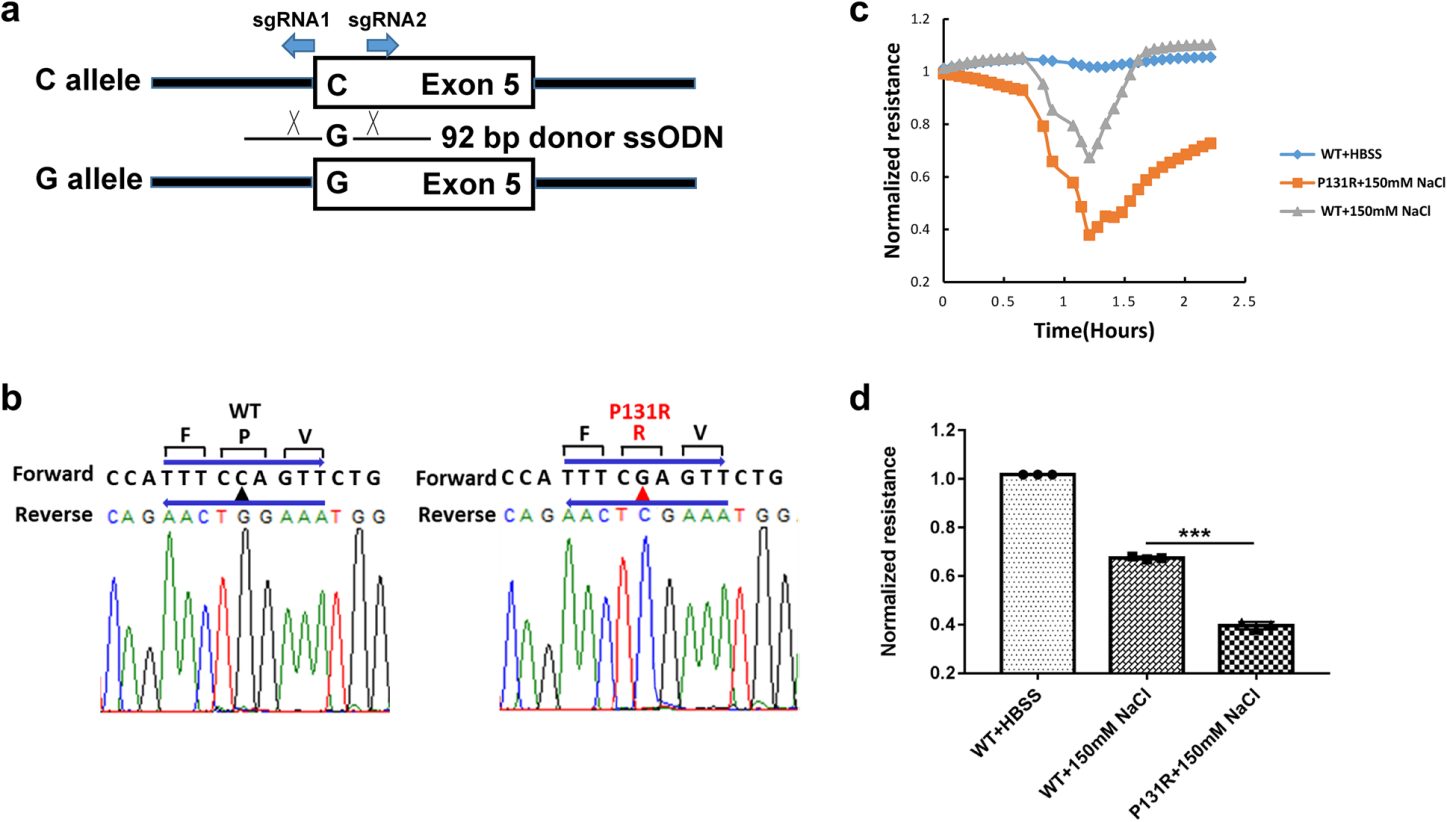
Construction and function of Slc26a3 P131R genetic variant on murine colonic epithelial (CMT-93) cells. **a** Generation of murine CMT-93 cells carrying the P131R SNP using the clustered regularly interspaced short palindromic repeats/Cas9 system. Outline of the targeting strategy to generate knock-in cell lines using a pair of composite Cas9n–sgRNA expression vectors and a donor single-stranded DNA oligonucleotide (ssODN) targeting the wild-type allele **c** in the Slc26a3 gene. **b** Results of Sanger sequencing for confirming the construction of P131R SNP on CMT-93 cells by CRISPR/Cas9. **c** CMT-93 cells were seeded and cultured overnight in growth medium, the monolayers were starved from serum for 2 h, then treated with 150 mM of NaCl. Representative figure of trans epithelial electric resistance (TEER) data from ECIS analysis of wild-type CMT-93 cells, Caco-2 cells containing the rs386833481(c.392C>G; p.P131R) SNP generated by CRISPR/Cas9 knock-in CMT-93 cells. **d** P131R Slc26a3 in CMT-93 cells significantly decreased the 150 mM NaCl-induced decrease in TEER value compared with WT CMT-93 cells. *P < 0.05, **P < 0.01, ***P < 0.001

To lay the foundation for in vivo experiments we investigated the role of P131R-Slc26a3 in murine epithelial barrier function. We adapted a similar HDR-mediated modification strategy using the CRISPR/Cas9 system to edit the Slc26a3 gene sequence in a murine colonic epithelial cell line (CMT-93, Fig. 6a). CMT-93 cells were transiently transfected with the mixture of two sgRNA constructs and an ssODN. Sanger sequencing assay (Fig. 6b) validated the construction of P131R-Slc26a3. To characterize the role of P131R-Slc26a3 in murine epithelial barrier function, we measured TEER in P131R-Slc26a3 and WT CMT-93 cells. Upon 150 mM NaCl treatment, P131R-Slc26a3 cells showed lower TEER values compared with WT cells (Fig. 6c). The maximum TEER inductions were 0.38 ± 0.01 in P131R-Slc26a3 vs. 0.67 ± 0.02 in WT (P < 0.001) (Fig. 6d). Notably, similar results were obtained when we constructed P131R-Slc26a3 and analyzed effect of this SNP on permeability in murine epithelial cells.

In summary, variant P131R-SLC26A3 increases the epithelial permeability and disrupts function of SLC26A3 through two distinct molecular mechanisms: (a) decreasing SLC26A3 expression through ubiquitination pathway and (b) disrupting a key interaction with ZO-1/CFTR, thereby increasing the epithelial permeability and induced epithelial barrier dysfunction.

## Discussion

In this study, we employed the CRISPR/Cas9 genomic editing tool to create human colonic epithelial Caco-2 cells containing the SLC26A3 SNP rs386833481 (c.392C>G; p.P131R) and then reverted the SNP back to its wild type sequence to investigate its effects on intestinal epithelial cell permeability. SNP rs386833481 was identified in patients with congenital chloride diarrhea (CCD), but its functional consequence was unknown. We have provided several lines of solid evidence that SNP rs386833481 caused the increased permeability in intestinal epithelial cells, indicating it is a likely causative mutation for diarrhea. We have also demonstrated that this mutation caused increased ubiquitination mediated degradation of SLC26A3, leading to decreased protein levels of SLC26A3. Our findings, along with other reports [27], demonstrated that SLC26A3 overexpression enhances intestinal epithelial cell barrier function and may explain why SNP rs386833481 mutation caused increased intestinal epithelial cell permeability. Our study is the first to supply the evidence that SLC26A3 SNP rs386833481 (c.392C>G; p.P131R) is a likely causative mutation for diarrhea and has also provided a molecular mechanism underlying this observation.

Here, we investigated the influence of P131R-SLC26A3 on the epithelial barrier, and the mechanisms of regulation in human colonic epithelial cells (Caco-2). Functional analysis showed that P131R-SLC26A3 was associated with increased dysfunction of the epithelial barrier induced by TNF-α (Fig. 2) and osmotic stress (Fig. 3a), while overexpression of SLC26A3 protected the epithelial barrier against TNF-α (Fig. 2). Moreover, P131R-SLC26A3 was involved in the [Cl^−^]_i_ decrease induced by Tenidap (Fig. 3b). Correction of P131R-SLC26A3 prevented the NaCl-mediated alteration of epithelial barrier (Fig. 4) and TJ protein to a certain extent compared with the normal control (Fig. 5a), suggesting that P131R-SLC26A3 might be critical for development of chronic diarrhea diseases caused by impaired epithelial barrier associated with disruption of TJ proteins. We further presented two mechanisms through which chronic diarrhea-risk-associated variant at 7q31.1 lead to increased dysfunction of the epithelial barrier by lower levels and activity of SLC26A3: (a) P131R-SLC26A3 reduced SLC26A3 expression through an enhanced ubiquitination mediated degradation pathway, and (b) a disrupted interaction with ZO-1/CFTR protein (Fig. 5), resulting in increased epithelial permeability and induced epithelial barrier dysfunction. Both mechanisms point to reduced function of SLC26A3 as a mechanism for disease pathogenesis.

We also recreated this SNP and investigated its influence on the function of epithelial barrier in murine colonic epithelial cells (CMT-93). The result similarly indicated that P131R-Slc26a3 caused increased intestinal epithelial cell permeability induced by osmotic stress (Fig. 6). Our ongoing studies are pursuing recreation and correction of this point mutation in an in vivo mouse model by the AAV-CRISPR system to evaluate its utility for therapeutic development in chronic diarrhea.

## Conclusions

In conclusion, our work using state of the art in vitro approaches has demonstrated that P131R-SLC26A3 (rs386833481) is a causal mutation in CCD, and correction of this SNP or increasing SLC26A3 function could be therapeutically beneficial for chronic diarrhea diseases. This is the first report defining function of the known SLC26A3 genetic variant. We corrected that variant, the mutant P131R allele, using the CRISPR/Cas9 mediated homologous recombination, and demonstrated restored normal epithelial barrier functionality of the corrected allele in the human colonic epithelial cells. Together with previous studies, which efficiently deliver the CRISPR components in vivo [28–31], this work provides a potential strategy for future gene therapy in patients with chronic diarrhea disease. Thus, our study is important in the elucidation of functional and biological consequences of SLC26A3 rs386833481, a likely therapeutic target in congenital chloride diarrhea, and with applicability to complex IBD, which exhibits reduced SLC26A3 expression and harbors some SNPs in its SLC26A3 [32, 33].

## Methods

### Cell culture

The Caco-2 cells (ATCC^®^ HTB-37™) and CMT-93 cells (ATCC^®^ CCL-223™) were obtained from ATCC. Caco-2 cells, which are human colorectal adenocarcinoma epithelial cells, were maintained in ATCC-formulated Eagle’s Minimum Essential Medium (EMEM, Catalog#: 30-2003), supplemented with 20% fetal bovine serum (Catalog#:S11150, Atlanta Biologicals, GA, USA) and 100 U/ml penicillin/streptomycin (Catalog#: 15140122, Thermo Fisher, Waltham, MA, USA). CMT-93 cells, which are murine colonic epithelial cells, were maintained in ATCC-formulated Dulbecco’s Modified Eagle’s medium (DMEM, Catalog#: 30-2002), supplemented with 10% FBS and penicillin/streptomycin. All cells were cultured at 37 °C in a humidified atmosphere of 5% CO_2_, 95% air. Cells from each primary flask were detached with 0.25% trypsin–EDTA (Catalog No. 25200056, Thermo Fisher, Waltham, MA, USA), re-suspended in fresh culture medium, and seeded into 6-well plates for the following experiments.

### CRISPR target sequence design

Guide sequences for CRISPR/Cas9 gene editing were designed as previously detailed [34], chemically synthesized, and RNase-Free HPLC purified by Integrated DNA Technologies

(Coralville, IA, USA). Single-strand ODN was chemically synthesized and standard desalted by Integrated DNA Technologies (Coralville, IA, USA). All sequences are listed in Table 1.

**Table 1:** Primer sequence for sgRNA cloning

Primer name	Primer sequence, 5′–3′
Human sgRNA1_T	CACCGAATTAACAGTGGGTGAATCG
Human sgRNA1_B	AAACCGATTCACCCACTGTTAATT
Human sgRNA2_T	CACCGCCGATTCTGAGTATGATGGT
Human sgRNA2_B	AAACACCATCATACTCAGAATCGG
Human sgRNA1a_T	CACCGATAAGACCATATAAAATGAC
Human sgRNA1a_B	AAACGTCATTTTATATGGTCTTAT
mouse sgRNA1_t	CACCGAGATAACCAGAGGTAAATGC
mouse sgRNA1_b	AAACGCATTTACCTCTGGTTATCT
mouse sgRNA2_t	CACCGCCAGTTCTGAGTATGATGGT
mouse sgRNA2_b	AAACACCATCATACTCAGAACTGG
Human ssODN	GAAACTGCTCCTGAAACTGCTAGTCCCACCATCATACTCAGAATCCGAAACGGACCTAATTAACAGTGGGTGAATCGTCGTCAGTATATGCCTCTCTAAAGCAC
Human ssODN-a	GAAACTGCTCCTGAAACTGCTAGTCCCACCATCATACTCAGGATAGGCAACGGACCTAATTAACAGTGGGTGAATCGTCGTCAGTATATGCCTCTCTAAAGCACATTGTCTTTCAACCACAGAATAAGACCATATAAAATGACTCGCAAGGCTGGGCGTGGTGGCTCACG
mouse ssODN	GTAACGACAACTCCCACCATCATACTCAGAACTCGAAATGGACCTAGATAACCAGAGGTAAATGCTCGTCAGTAGGTGCCTCCCTACGCCCG

We designed 2 guide RNAs (a forward and a reverse) that flank the SNP and a unique ssODN for a human epithelial cell line (Human sgRNA1, Human sgRNA2 and Human ssODN) and for a mouse epithelial cell line (mouse sgRNA1, mouse sgRNA2 and mouse ssODN), respectively. Each gRNA has a Top and Bottom oligo for cloning. For reverting the SNP to WT, we used different Forward sgRNA and a novel ssODN (Human sgRNA1a, Human sgRNA2 and Human ssODN-a) that code for the same amino acids as of WT SLC26A3, but use unique codons for the F-P-I triAA sequence flanking the wild-type Proline that was changed to Arginine and now back to Proline.

### Plasmid construction

Sense and antisense oligonucleotides for each sgRNA were annealed and inserted into a BbsI site of the pX462 plasmid expressing Cas9/gRNA scaffold [35]. pSpCas9n (BB)-2A-Puro (PX462) V2.0 was a gift from Feng Zhang (Addgene plasmid #62987). Cloning of annealed oligonucleotides was confirmed by Sanger sequencing analysis using the following primer: pLKO.1.5 FW, 5′-GACTATCATATGCTTACCGT-3′ (Lot: 14868230).

### SNP models in human Caco-2 cells and murine CMT-93 cells

A total of 200,000 cells were seeded in 6-well plate overnight in the regular growth medium, so that they would be 80–90% confluent at the time of transfection. One hour prior to transfection, media was removed and 750 µl of pre-warmed reduced serum OptiMEM media (Catalog#: 31985070, Thermo Fisher, Waltham, MA, USA) was added to each well. Transfection was performed using Lipofectamine 3000 and P3000 reagent (Catalog#: 3000015, Thermo Fisher, Waltham, MA, USA). For each well, 5 µl of P3000 reagent was diluted in 125 µl OptiMEM with pX462-gRNA plasmids (500 ng) and the donor plasmid (500 ng) containing a synthesized sequence for SNP. 5 µl of Lipofectamine 3000 was diluted in 125 µl OptiMEM and, after 3 min, it was added to the mixture of DNA and P3000 reagent. The complete mixture was incubated 15 min before being added to cells. After 6 h, the media was changed to 2 ml complete medium. GFP plasmid was used to monitor transfection efficiency. The puromycin concentration used for SNP selection was determined prior to cell selection by measuring cell sensitivity (1 µg/ml for Caco-2 cells and 2 µg/ml for CMT-93 cells). Puromycin selection was initiated 24 h post transfection for 72 h until WT control cells were all dead. Then transfected cells were cultured in regular medium with 0.1 µg/ml puromycin for 4-7 days and harvested for Taqman genotyping and sequence analysis.

### Sanger sequencing

Genomic DNA (gDNA) was extracted from puromycin selected cells using the Gentra Puregene Cell Kit (Catalog#: 51306, QIAGEN, Toronto, Canada) according to the manufacturer’s instruction. 50 ng of the isolated genomic DNA was used as template to amplify DNA by Platinum Taq (Catalog#: 10966-026, Thermo Fisher, Waltham, MA, USA) PCR. The amplicon DNA after PCR was verified by 2% agarose gel and gel purified using the Nucleospin Gel and PCR Clean-up kit (Catalog#: 740609.250, Clontech, Mountain View, CA, USA). The concentration and purity of DNA was determined by measuring absorbance at 260 and 280 nm using Take3 microspot plate reader (BioTek), and the nucleotide sequence of individual colonies was determined by sequencing using the following primer: Human SLC26A3_R: 5′-TCCCAAAGTGCTGGGATTAC-3′ (Lot: 162860013); Mouse Slc26a3_R: 5′-TACTGATGCAGCCACCATTAC-3′ (Lot: 190941198).

### ViiA7 TaqMan SNP genotyping assay

Genomic DNA (gDNA) was extracted from puromycin selected cells using the Gentra Puregene Cell Kit, according to the manufacturer’s instruction. The TaqMan fluorescently labeled probes (Catalog#: 4351379, Thermo Fisher, Waltham, MA, USA) targeting the studied rs386833481 SNP and genotyping Master Mix (Catalog#: 4371355, Thermo Fisher, Waltham, MA, USA) were used for DNA amplification in the ViiA7 Sequence Detection System (Applied Biosystems, USA). Genotyping was performed blinded to sample status. A non-template reaction (using water instead of DNA) was used as negative control and a sample of known genotype was used as positive control.

### Measurement of TEER (ECIS assay)

Cellular barrier properties (Transepithelial electric resistance, TEER) were measured using an electrical cell-substrate impedance sensing system (ECIS Ztheta; Applied Biophysics, Troy, NY, USA). Caco-2 or CMT-93 cells were seeded onto array chambers containing 40 gold electrodes per well (8W10E+, Applied Biophysics) pretreated with 10 mM cysteine and coated with fibronectin (20 µg) according to the manufacturers’ specifications. The experiments were initiated when the cells reached confluence, as determined by a capacitance of 10 nF at 32,000 Hz. Caco-2 or CMT-93 cells were starved for 2 h, then treated with 100 ng/ml of TNF-α (Catalog#: 210-TA and 410-MT-010, R&D Systems Inc., Minneapolis, MN, USA) or 150 mM NaCl (Catalog#: IB07072, IBI Scientific, Peosta, IA,USA). The data are presented as normalized resistance vs. time at 500 HZ. Resistance was averaged over the 40 electrodes per chamber and normalized so the time 0 resistance was 1.0.

### In vitro cell permeability assays

In vitro cell permeability assays were carried out according to the protocol of the CHEMICON In Vitro Vascular Permeability Assay kit (Catalog#: ECM644; Millipore, Billerica, MA, USA). Briefly, cells (1 × 10^5^) were seeded into the culture inserts of permeability chambers that were coated with collagen and incubated at 37 °C until a monolayer was formed. After the cells were starved from serum for 1 h with Hank’s balanced salt solution (HBSS), then treated with HBSS (control) or 150 mM of NaCl in HBSS. Cells were incubated for another 30 min at 37 °C, followed by addition of 75 μl of FITC-Dextran to each insert for 20 min at room temperature (RT), and then 100 μl of the solution in the bottom chamber was transferred to a black 96-well opaque plate. Absorbance at 485 and 535 nm was measured in a TriStar Multimode Reader (LB 941, Berthold Technologies GmbH & Co. KG, Bad Wildbad, Germany). Reagent control wells were treated with HBSS only. Blank inserts without plated cells were also included as controls.

### Intracellular Cl^−^ measurements in Caco-2 cells

The [Cl^−^]_i_ was measured in Caco-2 cells using MQAE (Catalog#: E3103, Thermo Fisher, Waltham, MA, USA), a Cl^−^ sensitive fluorescent dye, as previously described [20]. Briefly, Caco-2 cells were incubated with 10 mM MQAE for 30 min at 37 °C. Excess MQAE was removed by changing with fresh medium. The influence of SLC26A3 inhibitor (50 µM Tenidap, Catalog#: PZ0196, Sigma, St. Louis, MO, USA) on [Cl^−^]_i_ was determined after recording the basal fluorescence (350/460 nm excitation/emission) for 1–3 min, and measuring for a further 5–10 min after the addition of Tenidap. Two controls were performed: (1) DMSO (drug solvent) was added while the fluorescence was recorded; and (2) MQAE fluorescence without cells was recorded, and the Tenidap were added. No significant fluorescence changes were observed after performing both controls.

### Co-immunoprecipitation

For co-IP assays, Caco-2 cells were lysed on ice with non-denaturing lysis buffer for 1 min, then were scraped and gently transferred into a chilled microcentrifuge tube. The cells were mixed on a rotary mixer for 30 min at 4 °C. After centrifugation, the concentration of supernatants were assayed by BCA assay and incubated overnight at 4 °C with the SLC26A3 antibody (Catalog#: GTX34204, GeneTex, Irvine, CA, USA). We used 500 µg protein and 2 µg SLC26A3 antibody in 500 µl Lysis Buffer containing the protease inhibitor for WT, P131R and RWT at the same time. After antibody binding, add 25 µl of protein A/G Sepharose^®^ beads slurry to each tube and incubate for 1 h at 4 °C. The beads were then washed three times with 1× wash buffer, and the precipitates were eluted with sample buffer, separated by 7.5% SDS/PAGE, and analyzed by immunoblotting.

We had validated all antibodies in Caco-2 cells, CMT-93 cells and HCT116 cells, which are all colonic epithelial cells.

### Western blotting

Caco-2 cell lysates were collected on ice in RIPA buffer and isolated by centrifugation at 13,000 RPM for 10 min at 4 °C. Protein was quantified by Pierce BCA (Catalog#: 23225, Thermo Fisher, Waltham, MA, USA). 30 μg protein was boiled with sample buffer prior to loading on a polyacrylamide gel. SLC26A3 antibody diluted 1:1000 in TBS-T + 5% milk (Catalog#: GTX34204, GeneTex, Irvine, CA, USA), CFTR antibody diluted 1:500 in TBS-T + 5% milk (Catalog#:sc-376683, Santa Cruz Biotechnology, Dallas, TX, USA), ZO-1 antibody diluted 1:1000 in TBS-T + 5% milk (Catalog#: GTX108613, GeneTex, Irvine, CA, USA), GAPDH antibody diluted 1:2000 in TBS-T + 5% milk (Catalog#: Sc-25778, Santa Cruz Biotechnology, Dallas, TX, USA). Goat anti-rabbit HRP antibody diluted 1:10,000 in TBS-T + 5% milk (Catalog#: PI-1000, Vector Biolabs, Malvern, PA, USA) and horse anti-mouse HRP antibody diluted 1:10,000 in TBS-T + 5% milk (Catalog#: P1-2000, vector Biolabs Malvern, PA, USA) were used to visualize westerns. Bands were visualized by ECL (Pierce ECL Western Blotting Substrate, Catalog#: 32106, Thermo Fisher,Waltham, MA, USA) with a FluorChem M Imager (ProteinSimple, San Jose, CA, USA) and quantified by AlphaView Software SA, v.3.4.0.0.

### Statistical analyses

Statistical analyses were carried out using the Sigma Stat (ver.4.0, SysTest Software, Inc., San Jose, CA). All data were expressed as mean ± SD (standard deviation) of at least three independent experiments. Two group comparisons were done by an unpaired Student’s t test. Differences between groups were considered statistically significant at *P* < 0.05.

## Data Availability

Not applicable.

## Abbreviations

SLC26A3: solute carrier family 26 member 3
CCD: congenital chloride diarrhea
SNP: single nucleotide polymorphism
ssODN: single-stranded DNA oligonucleotide
ECIS: electrical cell-substrate impedance sensing system
